# First record of the genus *Gratia* Thomas (Ephemeroptera, Baetidae) from China with the description of a new species

**DOI:** 10.3897/zookeys.478.8995

**Published:** 2015-01-28

**Authors:** Weifang Shi, Xiaoli Tong

**Affiliations:** 1 Department of Entomology, College of Natural Resources & Environment, South China Agricultural University, Guangzhou 510642, China

**Keywords:** Mayfly, *Gratia*, new species, Oriental Region, Eastern Himalayas, Tibet

## Abstract

A new species of Baetidae, *Gratia baibungensis* **sp. n.**, is described and illustrated based on nymphal stage collected from the southeastern Tibet (Xizang) and the genus is reported for the first time from China. This new species can be readily differentiated from its congeners by the absence of a protuberance on the posterior margin of the abdominal tergum X, glabrate simple submarginal setae on the labrum, and the posterior margin of sterna VI–IX having much longer spatulate setae.

## Introduction

The Eastern Himalayas is situated between the west of central Nepal and east of Myanmar, through southeastern Tibet in China to northeastern India. The area has been declared as a biodiversity hotspot (Myers et al 2000, CFPF 2005). Historically, reports of freshwater macroinvertebrate fauna from this region are abundant (Allen et al 2010). However, in southeastern Tibet, as a part of the Eastern Himalayas, the aquatic insect fauna (mayfly in particular) remains rarely studied. You (1987) reported 13 mayfly species in Tibet, of which only 4 species were collected from the southeastern Tibet. Since then, the mayfly fauna of this region has received little attention.

In 2010, we conducted an exploration on the aquatic insect fauna in southeastern Tibet. The preliminary results revealed that mayflies have high species diversity, especially in Baetidae. Except for some species which are probably endemic to this region, most of mayfly fauna elements belong to the Indo-Malayan ecozone.

The genus *Gratia* was originally erected by Thomas (1992) based on the nymphal stage from Chiang Mai, Thailand, with *Gratia sororculaenadinae* Thomas as the type species. Later, Boonsoong et al (2004) found a second species of the genus, *Gratia narumonae* Boonsoong & Thomas, from Thailand and revised the generic concept. The present study deals with an undescribed species of the genus *Gratia* collected from southeastern Tibet.

## Materials and methods

Nymphs were collected by kick-net from the riffles with cobble and gravel substrates in a 2^nd^-order shallow stream (approximately 1 m wide) at the Baibung Town, Medog County, southeastern Tibet. The specimens were directly placed into vials with 95% ethanol in the field and transferred into glass vials with 85% ethanol in the laboratory for preservation. Some were dissected under the stereomicroscope and were mounted on slides in Hoyer’s solution for examination and illustration. Mounted structures were examined and photographed under the microscope with a digital camera attached, and the images subsequently processed with Adobe Photoshop CS6. Type specimens are housed in the Collection of Aquatic Insects and Soil Animals, Department of Entomology, College of Natural Resources and Environment, South China Agricultural University, Guangzhou, China. The comparative specimens of *Gratia sororculaenadinae* and *Gratia narumonae* were provided by Dr Boonsoong of Kasetsart University, Thailand.

## Taxonomy

### Key to mature nymphs of *Gratia* species

**Table:** 

1	Posteromedial dorsal protrusion presents on abdominal terga I to IX, submarginal setae on labrum simple and glabrate [China]	***Gratia baibungensis* sp. n.**
–	Posteromedial dorsal protrusion presents on abdominal terga I to X	**2**
2	All but the innermost two submarginal setae on labrum branched, second segment of labial palp with an inner-apical lobe [Thailand]	***Gratia narumonae* Boonsoong & Thomas**
–	All but the innermost two submarginal setae on labrum feathered, second segment of labial palp without an inner-apical lobe [Thailand]	***Gratia sororculaenadinae* Thomas**

#### Gratia baibungensis sp. n.

Taxon classificationAnimaliaEphemeropteraBaetidae

http://zoobank.org/B4B9E4BB-FD13-47BB-8315-D96F4196B0EA

##### Material examined.

**Holotype.** 1 mature nymph in ethanol, China, Tibet, Medog County, Baibung Town (29°14.65'N, 95°10.59'E, alt. 860m), 29.ix.2010, coll. Xianfu Li.

##### Paratypes.

2 nymphs on slides and 2 nymphs in ethanol, same data as holotype.

##### Other material examined.

*Gratia sororculaenadinae* Thomas: 3 mature nymphs, Thailand, Chiang Mai, Mae Hlang Stream, 16.i.2007; *Gratia narumonae* Boonsoong & Thomas: 3 mature nymphs, Thailand, Loei Province, Tarn Sawan waterfall, 12.ii.1999.

##### Description.

**Mature nymph** (Fig. 2a). Body length 4–4.5 mm, cerci slightly longer than body length, terminal filament only one segment.

**Figure 1.**
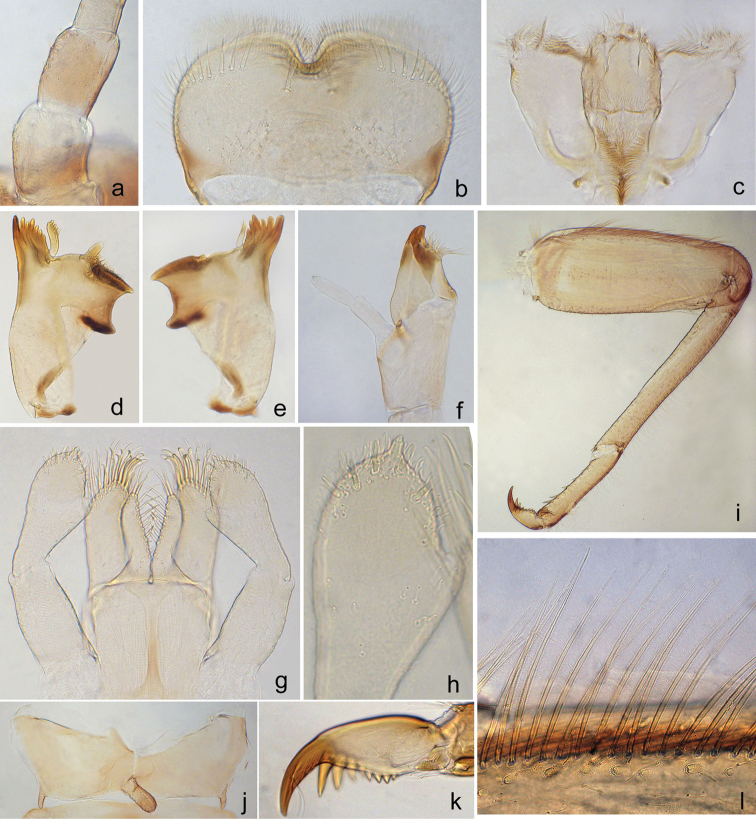
*Gratia baibungensis* sp. n. **a** antennal scape and pedicel **b** dorsal view of labrum **c** hypopharynx **d** left mandible **e** right mandible **f** left maxilla **g** labium **h** ventral view of labial palpus **i** foreleg **j** hindwing pad **k** hind claw **l** ciliate bristles on dorsal margin of femur.

***Head*.** Capsule yellowish-brown with transverse irregular markings on vertex and frons. Antennae approximately 1.5 times the width of head; dorsal surface of scape and pedicel scatter with fine setae, and with 3–4 and 2–3 scale-like setae respectively (Fig. 1a). Labrum (Fig. 1b) rectangular, approximately 2.0 times wider than long; anteromedial notch deep with a small rounded lobe at the base, and each side with one medial long seta and a row of 7–8 robust, simple and glabrate submarginal setae sublaterally, fine and simple setae scattered posteriorly; ventrally bordered with feathered setae along the anterior margin and a distomedial arc of very fine setae. Left mandible (Fig. 1d): incisors fused with 7 denticles, prostheca robust with 5 blunt and 3–4 acute denticles apically. Right mandible (Fig. 1e): incisors fused with 6 or 7 denticles, inner incisor margin smooth without fine setae, prostheca with denticles apically and distinctly slender than the one on the left mandible, edge between prostheca and molar smooth with no serration, molar plated-like. Hypopharynx with lingua rounded and superlinguae broadly truncate, covered with abundant fine setae (Fig. 1c). Maxillae (Fig. 1f) with one canina and three dentisetae on crown of galealacinia, a row of 4 long basal setae and one short bristle-like hump seta on basis of galealacinia; maxillary palpus 2-segmented and subequal in length, terminal segment with a small tip at apex and slender than basal segment. Labium (Fig. 1g): glossae slightly shorter than paraglossae, with a row of 9 stout setae along the inner margin dorsally and 2 long robust blunt setae at the apex; paraglossae approximately 2.0 times wider than glossae, with two rows of setae ventrally and 2 stout acute setae along the inner margin dorsally; labial palpus 3-segmented, terminal segment conical with a distinctive tip at apex (Fig. 1h); the 2^nd^ segment with an inner-apical lobe and an oblique row of 2–3 setae dorsally, the articulation between the 2^nd^ and terminal segments obscure; dorsal surface with numerous pores on the 1^st^ segment.

***Thorax*.** Coloration pale brown with indistinct darker patterns. Surface of pronotum with four blunt tubercles. Metanotum with a finger-like protuberance medially (Fig. 1j). Hind wing pads reduced, approximately 2 times longer than wide (Fig. 1j). Legs (Fig. 1i) yellow brown with darker markings. Femora of all legs with a regular row of multilaterally ciliate bristles along dorsal margin, approximately 1/2 as long as femur width (Fig. 1l); stout short submarginal scale-like setae present; villopore present. Tibiae subequal to femur in length; irregular rows of simple fine setae present on the dorsal margin, with length subequal to the width of tibia, submarginal stout setae present. Tarsi half the length of tibia, with irregular row of sparse fine simple setae on dorsal margin and 5–6 robust point setae on ventral margin increasing in length towards the apex, tarsus of all leg without long ventral subapical bristle. Claw with one row of 8–9 denticles and a pair of bowed subapical bristles (Fig. 1k). All legs lack coxal gills.

**Figure 2.**
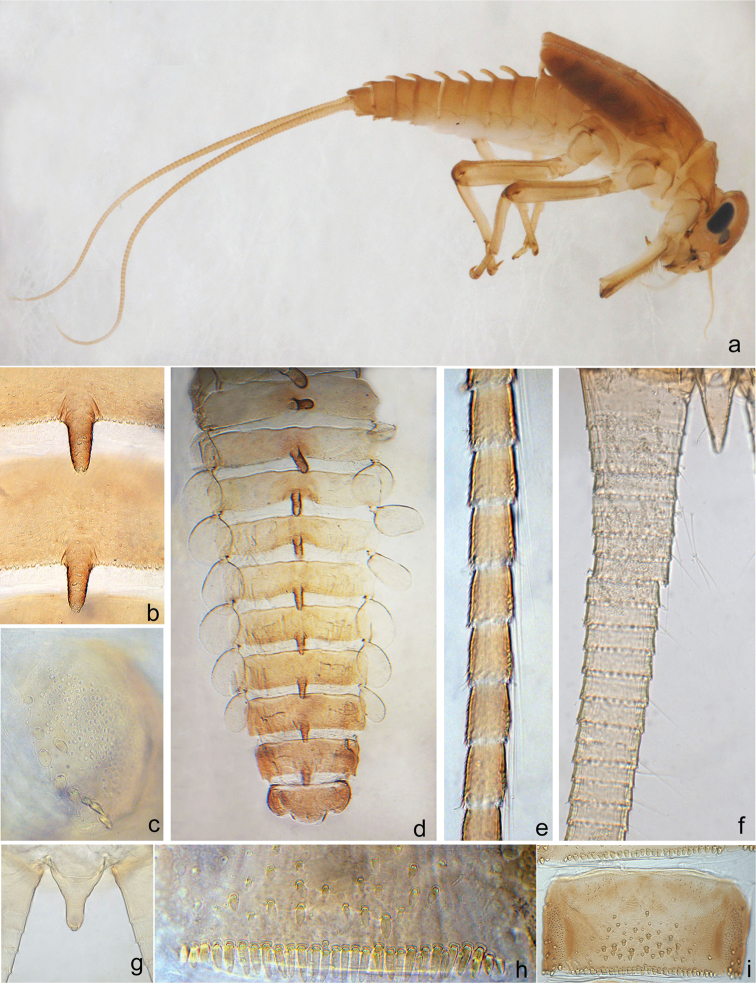
*Gratia baibungensis* sp. n. (**a–e, g, h**), *Gratia narumonae* (**f**) and *Gratia sororculaenadinae* (**i**). **a** habitus of lateral view **b** protrusion on abdominal terga VI–VII **c** paraproct **d** abdominal terga I–X **e** inner marginal bristles on cerci **f** inner marginal bristles on cerci **g** terminal filament **h** posterior margin of abdominal sterna VIII **i** sterna VII–VIII.

***Abdomen*.** Generally yellowish-brown. Mediodorsal posterior margin of terga I–IX each with a finger-like protuberance, successively decreasing in length backwardly (Figs 2a, d): length of protuberance subequal to the tergum length on segments I–IV, and approximately one third of tergum length on segment IX; surface of protuberance scattered with scale-like setae (Fig. 2b). Terga surface with scattered scale-like and fine setae, posterior margin with blunt denticles. Surface of sterna scattered with round scale-like setae and each sternum with a pair of friction pads on anterolateral area; posterior margin on sterna VI–IX each with a row of continuous long spatulate setae which length approximately 3–4 times the width in female nymphs (Fig. 2h) and 2–3 times the width in male nymphs. Paraproct (Fig. 2c) with numerous pores and fine bristles on the surface and 9–14 scale-like setae along the inner margin. Gills on segments I–VII, oval and untracheated, surface scattered with numerous pores, margin smooth with blunt and fine simple setae. Median caudal filament reduced to one segment (Fig. 2g), each segment of cerci on inner margin with 1–4 (increasing in number towards terminal) swimming bristles (Fig. 2e).

##### Adult.

Unknown.

##### Etymology.

This new species is named after Baibung, the small town near the type locality.

##### Distribution.

China (southeastern Tibet).

##### Remarks.

Comparison with Thai specimens of two *Gratia* species (Table 1) shows that this new species can be readily distinguished from other members of the genus by the absence of a protuberance on tergum X. In appearance, the new species is most similar to *Gratia narumonae* Boonsoong & Thomas, but it can be readily distinguished from the latter by the following characters:

1. all dorsal submarginal setae on labrum are glabrate and simple (vs. all but two innermost setae are branched and fimbriate in *Gratia narumonae*);
2. posterior margin on sterna VI–IX each with a row of continuous long spatulate setae approximately 2–4 times longer than wide (Fig. 2h), but only 1.5–2 times in *Gratia narumonae* (cf. Fig. 2i);
3. gill margin with blunt setae, absent in *Gratia narumonae*.

**Table 1.** Comparison of morphological characters of the three *Gratia* species.

Character	*Gratia baibungensis* sp. n.	*Gratia narumonae*	*Gratia sororculaenadinae*
Body length	4–4.5 mm	6.4 mm	4.7–5.8 mm
Number of scale-like setae on scape and pedicel	5–7	7–9	Approx. 30
Number of submarginal setae on labrum	1+(7–8)	1+(10–12)	1+(15–16)
Shape of submarginal setae on labrum	Simple and glabrate	Branched	Feathered
Width/length ratio of labrum	1.96	1.92	1.93
Inner-apical lobe on 2^nd^ segment of labial palp	Present		Absent
Number of denticles on claw	8–9	8–10	7–9
Finger-like protuberance on terga	I–IX	I–X	
Ratio of scale-like setae length/width on posterior sterna VI–IX.	2.0–4.0	1.0–2.0	
Number of scale-like setae on the inner margin of paraproct	9–14	11–16	20–25
Bristles on inner margin of cerci	Each segment with 1–4 bristles (increasing in number towards terminal)*		

* Boonsoong et al (2004) stated that cerci are devoid of bristles in both *Gratia* species, but according to our examination of the specimens of the two Thai *Gratia* species provided by Dr Boonsoong, bristles on cerci could be observed under the microscope (Fig. 2f).

##### Habitat.

Nymphs of *Gratia baibungensis* sp. n. inhabit the riffles area with cobble and gravel substrates in a 2nd-order shallow subtropical stream (approximately 1 m wide). However, both Thai species are found clinging to rock surfaces of tropical cascades: *Gratia sororculaenadinae* lives in limimadicolous zones and *Gratia narumonae* is more abundant in petrimadicolous zones.

## Discussion

The genus *Gratia* resembles *Jubabaetis* Müller-Liebenau in appearance by the presence a regular row of ciliate bristles on the dorsal margin of the femur, the protuberance on the posterior margin of abdominal terga, and the terminal filament reduced to one segment (Müller-Liebenau 1980). However, the shield-like prolongation on the frontal margin of head and the unique mouthpart characters in *Jubabaetis* can be easily distinguished from those of *Gratia*. Similar ciliate bristles on the dorsal margin of the femur are also found in the genus *Acentrella* Bengtsson, but it can be easily separated from *Gratia* by the terminal segment of the labial palpus, which is widely rounded and slightly truncate, devoid of subapical bristles on claw, and the abdominal terga without mediodorsal protrusions.

In comparison with the related genera, the genus *Gratia* is most closely related to the genus *Baetiella* in nymphal stages by sharing many similar morphological characters (Table 2), but the peculiarity of *Gratia* is that the femur on the dorsal margin has a regular row of multilaterally ciliate bristles (unlike *Baetiella* whose femur on the dorsal margin bears a densely irregular row of glabrate bristles, especially in the basal area of femur). Boonsoong et al (2004) summarized the generic diagnosis of *Gratia*; in the present study, the nymphal generic diagnostic is modified for *Gratia* as follows:

1. submarginal setae on labrum branched and fimbriate or simple and glabrate;
2. hind wing pads reduced or vestigial;
3. terminal segment of labial palpus conical with a tip on apex;
4. femora on dorsal margin with a regular row of ciliate (feathered) bristles, tibiae and tarsi with irregular row of fine glabrate setae;
5. femoral villopore present;
6. claws with a pair of subapical setae;
7. abdominal terga I–X (sometimes on I–IX) each with a finger-like protrusion on posterior margin, sterna and paraproct with conspicuous scale-like setae;
8. terminal filament reduced to one segment, inner margin of cerci with sparse bristles.

**Table 2.** Summary of diagnostic characters of the genera *Gratia* and *Baetiella*.

Character	*Gratia*	*Baetiella*
Antennae length	Approx. 1.5 times width of head	
Scale-like setae on scape and pedicel	Present	Absent or present
Submarginal setae on labrum	Branched and fimbriate or simple and glabrate	Simple and glabrate
Shape of terminal segment of labial palpus	Conical with a tip on apex	Conical with or without a tip on apex
Hind wing pads	Vestigial	Vestigial or well developed
Metanotum with a protrusion medially	Present	Present or absent
Bristles on dorsal margin of femur	In a regular row with ciliate (feathered) bristles	In a dense and irregular row with glabrate bristles
Femoral villopore	Present	
Ventral subapical bristle on tarsus	Absent	
Claw with a pair of bowed subapical bristles	Present	
Posteromedial dorsal protrusion on terga	Single, on I–X (or I–IX)	Single or double or absent, at most on I–IX
Median caudal filament	One segment (conical)	One to multi-segment

## Supplementary Material

XML Treatment for Gratia baibungensis
